# Measurement of Autophagy Activity Reveals Time-Dependent, Bacteria-Specific Turnover during *Mycobacterium tuberculosis* Infection

**DOI:** 10.3390/pathogens12010024

**Published:** 2022-12-23

**Authors:** Naomi Okugbeni, André du Toit, Victoria Cole-Holman, Glynis Johnson, Ben Loos, Craig Kinnear

**Affiliations:** 1 DSI-NRF Centre of Excellence for Biomedical Tuberculosis Research, US/SAMRC Centre for Tuberculosis Research, Division of Molecular Biology and Human Genetics, Department of Biomedical Sciences, Faculty of Medicine and Health Sciences, Stellenbosch University, Tygerberg 7505, South Africa; 2 South African Medical Research Council Genomics Centre, Tygerberg 7505, South Africa; 3 Neuro Research Group, Department of Physiological Sciences, Faculty of Sciences, Stellenbosch University, Stellenbosch 7602, South Africa

**Keywords:** autophagic flux, autophagy turnover, biomarker, immunofluorescence microscopy, LC3B, p62, RAW 264.7, THP-1, tuberculosis, western blot

## Abstract

The intracellular pathogen, *Mycobacterium tuberculosis* (M. tb) uses various mechanisms to evade its killing. One of such is phagosomal damage and cytosolic translocation which is then targeted by the host’s bactericidal autophagy pathway. It is suggested that cytosolic translocation of M. tb is time-dependent, occurring at later time points of 48 to 72 h post-infection. It is, however, not known whether increased autophagic targeting correlates with these time points of infection. We investigated the time-dependent profile of autophagy activity through the course of M. tb infection in mammalian macrophages. Autophagy activity was inferred by the turnover measurement of autophagy markers and M. tb bacilli in THP-1 and RAW 264.7 macrophages. Over a period of 4 to 72 h, we observed highest autophagy turnover at 48 h of infection in M. tb-containing cells. This was evident by the highest turnover levels of p62 and intracellular M. tb. This supports observations of phagosomal damage mostly occurring at this time point and reveal the correlation of increased autophagy activity. The findings support the preservation of autophagy activity despite M. tb infection while also highlighting time-dependent differences in M. tb-infected macrophages. Future studies may explore time-dependent exogenous autophagy targeting towards host-directed anti-tuberculosis therapy.

## 1. Introduction

Tuberculosis (TB) is an infectious respiratory disease caused by the bacterium, *Mycobacterium tuberculosis* (M. tb) [1]. Despite the 2–4% decline in incidence rate per year, the number of yearly deaths remains high (1.6 million in 2021) [2]. This burden is attributed to co-morbidities with other immune-suppressing illnesses/behaviours, lack of rapid point of care diagnostics, lack of effective disease-monitoring measures, the rise of antibiotic-resistant strains and the need for more effective drugs. In a bid to alleviate the TB burden, research into the use of host-directed therapy (HDT) for TB treatment has seen significant growth within the last 10 years. This field explores the application of adjunctive pharmaceuticals towards strengthening the host immune system by targeting pathways modulated by the disease. In TB, HDT offers the advantage of bypassing the challenge of antibiotic resistance and reduces the incidence of toxicity observed with current TB antibiotics and consequently improves patient adherence [3]. The need for HDT in TB is crucial [4,5,6], however, the basic molecular and temporal mechanisms must be better understood, particularly regarding the pathogenesis of M. tb [3,7].

The significance of time as a major determinant of the outcome of biological experiments cannot be overemphasized [8,9,10,11]. Most biological processes are highly dynamic and are temporally regulated. These processes should therefore be studied over a period. Key interventions in the experiments can be used to decide on the frequency and number of time points to be studied, for example, the replicating rate of an infecting bacterium when studying a pathway in a host cell [8,10].

Upon infection, M. tb is first encounters alveolar macrophages and phagocytosed for impending lysosomal degradation. *Mycobacterium tuberculosis* can inhibit lysosomal killing through various mechanisms including damaging the phagosome and escaping into the cytosol [12,13,14,15,16,17]. This damage activates the host autophagy pathway [18,19,20].

Autophagy, a degradative process conserved in all eukaryotic cells, functions by forming an encapsulating double-membraned phagophore around excess or unwanted cytosolic material that delivers the cargo to the lysosome where it is degraded. Initially, autophagy was thought to be solely a means of survival in times of nutrient starvation and stress [18]. However, it is also now recognized to be a form of innate immune defense against intracellular pathogens as well [21,22].

Initial evidence for the role of autophagy in M. tb arose when Gutierrez and co-workers [23] observed that upon exogenous induction of autophagy, M. tb survival was reduced in infected host macrophage cells. Further observation has also revealed that autophagy evasion is one strategy used by M. tb to avoid host killing [24]. Research focusing on anti-tuberculosis HDT is now increasingly exploring how autophagy can be stimulated to ensure more efficient mycobacterial killing [25,26,27,28]. However, for such an approach to be successful, the dynamic interplay between autophagy activity and M. tb requires elucidation. For example, the time point during infection when phagosomal damage and autophagy activation occur is not clear, and whether there are cyclic periods of autophagy induction and evasion during infection. While some have observed cytosolic translocation of M. tb to only occur from 48 h post-infection onwards [17,29,30], a concomitant induction in autophagy at these specific time points have not been reported. Conversely, others have observed autophagic targeting at earlier time points [18,19], suggesting some level of phagosomal damage. A clearer understanding of the temporal dynamics of autophagy during M. tb infection will not only provide clearer understanding of M. tb pathogenesis in host cells [11] but also help in the development of anti-TB HDT.

Markers such as LC3B-II and/or p62 are routinely used to study this autophagy as LC3B lipidation has been shown to be a good marker that correlates well with autophagosome abundance [31,32,33,34]. P62, on the other hand, is a critical receptor protein that recruits cargo to the autophagosome and is subsequently being degraded. It has recently been shown that p62 levels respond rapidly to autophagy induction [32,33,34,35]. In M. tb infection, studies either use one autophagy marker [3,36,37,38,39,40,41,42,43,44] to track this process or several markers at a single time point [3,38,39,40,41,42,45,46] to investigate the autophagy induction capability of their protein/treatment of interest. These approaches do however have their limitations. For example, LC3B-II present on autophagosomal membranes is also present on phagosomes in a specialized type of phagocytosis termed LC3-associated phagocytosis (LAP) [47,48]. As such, for M. tb-infected cells, LC3B-II alone is not specific enough to study damaged-induced autophagy. Furthermore, autophagy activity or flux, i.e., the rate of protein degradation through the entire autophagy pathway, is typically measured to compare different treatment groups, while a comprehensive assessment of the autophagy activity profile over time has not yet been reported.

This scenario is made even more complex due to the cell heterogeneity. The activity level of basal autophagy is well reported to be cell dependent [49,50] but within one cell population, studies have also observed varying levels of autophagic activity [51]. This is particularly relevant in M. tb infection experiments as another layer of complexity is added when not all the cells in the infected cell pool contain bacteria. While increasing the multiplicity of infection (MOI) will increase the percentage of bacteria-containing cells, it also increases host cell death [52,53,54]. The heterogenous population of cells in the infection pool most often conceals biologically significant observations present in the minority population of bacteria-containing cells. Despite these concerns, there is a lack of studies that measure autophagic activity in the different subpopulations of infected cells.

This study therefore addressed these limitations and improved on previous M. tb-autophagy studies by measuring the progression of autophagy activity in uninfected and M. tb-infected macrophages subpopulations. This was achieved using multiple techniques and markers of autophagy over a series of time points post-infection. This time course approach provides more clarity on the autophagic flux-M. tb interplay during infection. In addition, some of the unconventional methods employed in this work may serve as a toolbox for further research to build upon.

## 2. Materials and Methods

### 2.1. Cell Lines

THP-1 cells (American Type Culture Collection, Manassas, VA, USA, ATCC-88081201) were grown and maintained in Roswell Park Memorial Institute (RPMI) medium (Sigma-Aldrich, Johannesburg, South Africa, R8758) while RAW 264.7 cells (ATCC TIB-71) were grown and maintained in Dulbecco’s Modified Eagle’s Medium (DMEM) medium (Lonza, Basel, Switzerland, BE12-604F). Both media were supplemented with 10% Fetal Bovine Serum (FBS) (Thermo Scientific, Johannesburg, South Africa, 10493106), and 1% Penicillin-Streptomycin (Lonza, DE17-602E). THP-1 cells were differentiated into macrophage-like cells (dTHP-1) by inducing with 162 nM Phorbol 12-Myristate 13-Acetate (PMA (Sigma-Aldrich, P8139) for 72 h before resting in PMA-free culture medium for 24 h before downstream experiments [55,56]. RAW 264.7 macrophages were seeded overnight with an FBS concentration of 2.5% to reduce cell multiplication while still maintaining viability over the time course experiment.

### 2.2. Bacterial Culture Conditions

The laboratory strain of M. tb, H37Rv was obtained through BEI Resources, NIAID, NIH: *Mycobacterium tuberculosis*, Strain H37Rv, NR-13648 and used for M. tb infection experiments. This strain was transformed with a mCherry-expressing plasmid (Addgene, Watertown, MA, USA, 24659; deposited by Tanya Parish). All culturing and infection experiments were performed in a Biosafety Level III facility. The non-pathogenic close relative of M. tb, *Mycobacterium smegmatis* mc2155 (M. smeg; ATCC 700084), was used as a non-pathogenic control bacterium in some infection experiments [57]. For stock solutions, bacterial cells were grown in Middlebrook 7H9 medium (Becton-Dickinson, Franklin Lakes, NJ, USA, 271310) supplemented with 10% Oleic acid Albumin Dextrose Catalase (OADC) (Becton-Dickinson, 212240), 0.2% glycerol, 0.05% Tween 80 and 50 µg/mL Hygromycin (for mCherry selection) under optimal growth conditions of 370C and shaking at 180 rpm. Stocks were frozen at an Optical Density of 600 nm (OD600) of approximately one.

### 2.3. Infection Experiments

For infection experiments, frozen stocks of bacteria were grown in a tween-free medium to OD600 of 0.5–0.8 (log phase) prior to infection [58,59]. Cells were spun at 2000 g for 10 min and resuspended in the appropriate host cell culture medium without antibiotics. To achieve single bacterial cells, bacterial cells were passed through a 21 G needle 20 times followed by 10 times through a 25 G needle. Thereafter, the culture was passed through a 5.0 µm filter and the resulting filtrate was quantified with an optical density spectrophotometer [58,59]. The filtrate was diluted appropriately and added as a monolayer suspension to host cells for a resulting Multiplicity of Infection (MOI) of two bacilli to one host cell or as indicated. Cells were incubated at 37 °C and 5% CO_2_ for four hours to allow for phagocytosis. Thereafter, macrophages were washed twice with the macrophage culture medium to remove extracellular bacteria and the infection was allowed to proceed for a total of 24, 48, and 72 h. At 4 h, a subset of the macrophages was lysed or fixed as the first time point. A parallel experiment was also carried out for uninfected macrophages. For each time point, Bafilomycin A1 (Sigma-Aldrich, B1793) was added to the culture medium at a final concentration of 100 nM for three hours prior to lysing or fixing of cells. 

### 2.4. Colony-Forming Units (CFU) Enumeration

At each time point after infection and Baf treatment, cells were lysed in 0.05% Sodium Dodecyl Sulphate (SDS), serially diluted and plated onto Middlebrook 7H11 agar plates (Becton-Dickinson, 212203) supplemented with 10% OADC. The plates were incubated at 37 °C, and the resulting colonies were counted after 21 days.

### 2.5. Western Blot Analysis

Equal amounts of whole cell lysates were extracted from THP-1 or RAW 264.7 infected with M. tb or as indicated. Lysates were run on SDS-PAGE gels (4–20% for LC3B-II and LAMP1; 10% for p62 and RAB7) and transferred to Polyvinylidene difluoride (PVDF) membranes (Thermo Scientific) using the iBlot II transfer system (Thermo Scientific). For immunoblotting, the membrane was first blocked for one hour with 5% BSA in Tris-Buffered Saline with Tween 20 (TBST) buffer. After blocking, the membrane was washed with TBST and incubated overnight at 4 °C with the following antibodies as indicated: anti-LC3B (Abcam, Cambridge, UK, Ab51520; 1:2000), anti-p62 (Abcam, Ab91526; 1:2000), anti-RAB7 (Sigma-Aldrich, R4779; 1:1000), anti-LAMP1 (Abcam, Ab24170, 1:2000), anti-Beta-actin (Abcam, Ab8227; 1:1000) or anti-GAPDH (Santa Cruz, sc-32233; 1:5000). The next day, the membrane was washed three times with TBST and subsequently incubated with the respective secondary antibody for one hour at room temperature: HRP-conjugated anti-rabbit (Cell Signaling, #7074; 1:5000) or HRP-conjugated anti-mouse (Cell Signaling, #7076; 1:5000). Thereafter, the membrane was washed three times with TBST and incubated with the Enhanced chemiluminescent (ECL) Clarity substrate (Bio-Rad, 1705061) for illumination using the ChemiDoc Imaging System (Bio-Rad, Hercules, CA, USA). Band intensities were calculated using the FIJI https://imagej.net/Fiji (Version 1.52p, accessed 20 July 2019) software and GAPDH or Beta-actin were used as loading controls as indicated.

### 2.6. Immunofluorescence Sample Preparation and Image Acquisition (RAW 264.7 Cells)

RAW 264.7 cells were seeded overnight in 8-well chambers prior to infection and Baf treatment. At each indicated time point post-infection, cells were washed with PBS and fixed with 4% paraformaldehyde solution in PBS for one hour at room temperature. Thereafter, cells were washed for 10 min with PBS and permeabilized with 0.2% Triton-X 100 in PBS for 10 min at room temperature. Subsequently, cells were washed in PBS and blocked with 3% BSA in PBS for two hours at room temperature. For antibody incubation, anti-LC3B (Abcam, Ab51520; 1:100) or anti-p62 (Abcam, Ab91526; 1:100) was diluted in 0.3% BSA in PBS and incubated with cells overnight at 40C. The next day, cells were washed for 30 min with 0.05% Tween 20 in PBS and subsequently incubated with anti-rabbit Alexa Fluor^®^ 488 (Abcam, Ab150077; 1:500) and Phalloidin (Thermo Scientific, A22287; 1:25) for 90 min at room temperature in the dark. Thereafter, the cells were washed with 0.05% Tween 20 in PBS for 30 min and then incubated with Hoechst 33342 nuclear stain (Thermo Scientific, 33342; 1:200) for 10 min at room temperature. After nuclear staining, cells were washed with PBS and mounted with DAKO mounting medium (Agilent, S302580-2) for cell preservation. Cells were imaged on a Carl Zeiss 780 PS.1 (Oberkochen, Germany) confocal microscope and the raw image stacks/series were acquired with a Plan-Apochromat 63x/1.4 Oil DIC M27 objective. The light sources were 405 nm, 488 nm, 561 nm, and 633 nm lasers with a GaAsP detector for emission detection. The laser power and master gain were selected for 405 nm (Hoechst), 488 nm (Alexa-Fluor 488), 561 nm (mCherry), and 633 nm (Phalloidin 647) to ensure an optimal signal/noise ratio with minimal pixel saturation. The track filters were set as follows: Hoechst; 410–497 nm, Alexa-Fluor 488; 499–579 nm, mCherry; 579–641 nm, Phalloidin 647; 645–747 nm.

### 2.7. Immunofluorescence (Confocal) Image Analysis

Confocal image analysis was performed in the Python programming language https://www.python.org/ (Version 3.7.3, accessed 5 August 2019) from the Anaconda 4.2.0 distribution https://www.anaconda.com/, accessed 5 August 2019. The following packages were used to script the image analysis pipeline: NumPy https://numpy.org/ (Version 1.16.4, accessed 5 August 2019), SciPy https://www.scipy.org/ (Version 1.3.0, accessed 5 August 2019), Sci-Kit Image https://scikit-image.org/ (Version 0.21.2, accessed 5 August 2019) OpenCV. https://opencv.org/ (Version 3.4.2, accessed 5 August 2019), Matplotlib https://matplotlib.org/ (Version 3.1.1, accessed 5 August 2019) and Panda https://pandas.pydata.org/ (Version 1.0.3, accessed 5 August 2019). 

### 2.8. Statistical Analyses

All results represent the mean ± SEM of at least three independent experiments. Western blot band intensities were quantified with FIJI and normalized to a housekeeping protein (GAPDH or Beta-actin as indicated). Turnover was calculated by subtracting control sample data from Baf treated data for western blot and puncta results and dividing control sample data by Baf treated data for bacterial enumeration results. Control and Bafilomycin sample groups were compared using multiple t-tests, Control samples and turnover values were compared over time using one-way ANOVA and Dunnett’s test for multiple comparisons. GraphPad Prism (http://www.graphpad.com/scientific-software/prism/) (Version 8.0.1, accessed 17 December 2019) was used for these statistical analyses and plotting graphs. *p* values of <0.05 were considered statistically significant with the following denotations: * *p* < 0.05, ** *p* < 0.01, *** *p* < 0.001 and **** *p* < 0.0001.

## 3. Results

### 3.1. Autophagy Activity Is Higher in M. tb-Infected THP-1 Macrophages Compared to Uninfected Macrophages at 24 and 72 h Post-Infection

**Figure 1:**
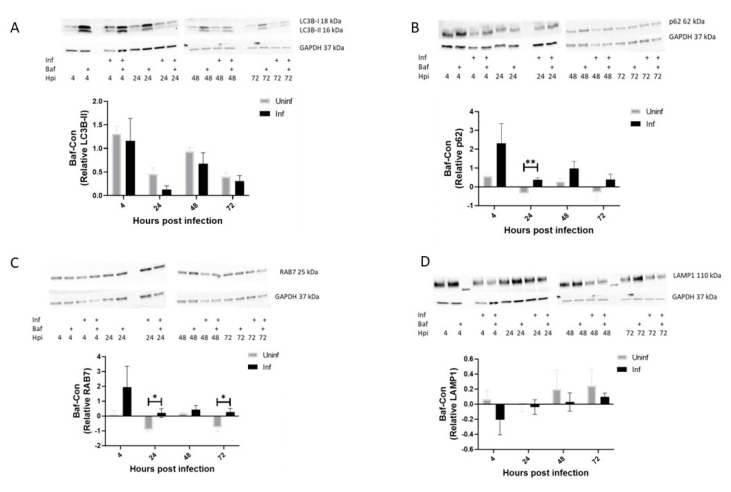
**Differential turnover of uninfected and M. tb-infected THP-1 macrophages.** THP-1 macrophages were infected with M. tb for 4, 24, 48 and 72 h. At each time point, proteins were extracted, and western blot was performed for LC3B (**A**), p62 (**B**), RAB7 (**C**) and LAMP1 (**D**). Turnover was calculated by subtracting control (Con) values from Bafilomycin treated (Baf) western blot expression values for the respective proteins at different time points. Data are represented as mean ± SEM from three independent experiments. (* *p* ≤ 0.05, ** *p* < 0.01 Multiple *t*-test). Uninf: Uninfected samples, Inf: Infected samples, Con: DMSO vehicle control, Baf: Bafilomycin A1 lysosomal inhibitor. X axis indicates time in hours and y axis indicates arbitrary units of expression of turnover values of the target protein relative to GAPDH expression.

To investigate the effects of infection on autophagy activity, we measured the turnover of four autophagyproteins: LC3B-II: a phagosome and autophagosome associated protein, p62- an autophagosome cargo receptor, RAB7- a marker of mature phagosomes and autophagosomes and LAMP1- a lysosome associated protein. We then compared this turnover between uninfected and M. tb-infected cells at 4-, 24-, 48- and 72-h post-infection. LC3B showed no significant differences at all time points (Figure 1A). This indicated that no increased level of autophagic degradation occurred in M. tb-infected cells, although a trend towards decreased levels was seen in the M. tb-infected cells when compared to uninfected macrophages.

Similar results were found for the selective autophagosomal marker p62, except at 24 h where a significant increase in turnover was observed in M. tb-infected macrophages (Figure 1B). This significance, however, was mostly likely due to the negative and generally lower values of turnover seen in uninfected macrophages at 24 and 72 h (Figure 1A–D). Next, we investigated the turnover of the mature phagosomal and autophagosomal associated protein RAB7 which showed a similar trend to p62 with significant differences at 24 and 72 h (Figure 1C). Lastly, as a negative control, we investigated the turnover of the lysosomal associated protein LAMP1. As expected, the turnover values had no statistically significant differences (Figure 1D). Taken together, these results showed that autophagosomal turnover differed in uninfected versus M. tb-infected mostly at 24 h post-infection. These results also introduce the possibility that at certain time points autophagic proteins could decrease in levels and not accumulate after Bafilomycin treatment either due to inherent regulation or from regulation of the housekeeping protein.

### 3.2. LC3B-II Induction in M. tb-Infected Macrophages Remains Stable over Time with Efficient Turnover at 4 and 48 h Post-Infection

**Figure 2:**
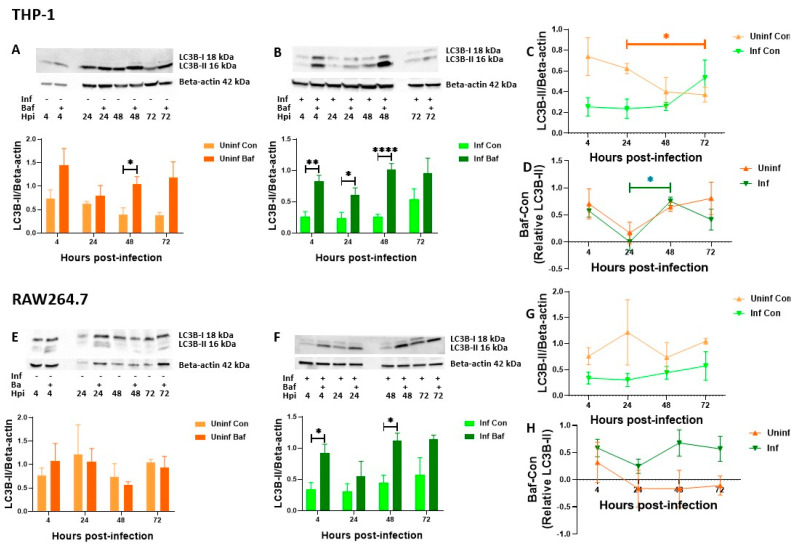
**LC3B-II protein levels and turnover in THP-1 and RAW 264.7 macrophages over time.** THP-1 and RAW 264.7 macrophages were infected with M. tb for 4, 24, 48 and 72 h. At each time point, proteins were extracted, and western blot was performed for LC3B. Expression levels for uninfected THP-1 and RAW 264.7 macrophages are depicted in (**A**,**E**) respectively while infected macrophages are depicted in (**B**,**F**). Turnover was calculated by subtracting control (Con) values from Bafilomycin treated (Baf) western blot expression values for the respective proteins at different time points. Control only expression is depicted in (**C**,**G**) while turnover values are depicted in (**D**,**H**). Data are represented as mean ± SEM from at least three independent experiments. (* *p* ≤ 0.05, ** *p* < 0.01, **** *p* < 0.0001 Multiple *t*-test (**A**,**B**,**E**,**F**), Two-way ANOVA with Tukey test for multiple comparison (**C**,**D**,**G**,**H**). Uninf: Uninfected samples, Inf: Infected samples, Con: DMSO vehicle control, Baf: Bafilomycin A1 lysosomal inhibitor. X axis indicates time in hours and y axis indicates arbitrary units of expression of turnover values of the target protein relative to Beta-actin expression.

Having established that uninfected and M. tb-infected macrophages differ in their autophagic activities at 24 and 72 h, we then followed these cells over time and compared their expression and turnover levels. These experiments were also performed in RAW 264.7 mouse macrophages to account for possible cell lines differences. Additionally, we changed our loading control protein to Beta-actin to investigate whether GAPDH regulation was responsible for negative turnover values observed in Figure 1B–D. We continued these experiments measuring only LC3B and p62 as RAB7 turnover values were similar to and correlated with p62. Also, RAB7 is not selective for damaged-induced autophagosomes in comparison to p62. Uninfected THP-1 macrophages showed a steady decrease with significance between 24 and 72 h (Figure 2A,C) while RAW 264.7 cells showed a relatively unchanged level of expression (Figure 2E,H).

On the other hand, LC3B in M. tb-infected cells displayed relatively stable levels over time in both THP-1 and RAW 264.7 (Figure 2B,C,F,G) suggesting no significant induction or change in degradation over time. To further resolve these observations, we investigated the LC3B turnover levels. It could then be ascertained that the protein levels of LC3B observed were in fact steady state levels due to a similar rate of induction and degradation. We observed significant autophagy turnover in infected macrophages at 4 and 48 h (Figure 2B,F) indicating efficient autophagic degradation was occurring. LC3B turnover in infected THP-1 macrophages, further displayed a significant increase from 24 to 48 h (Figure 2D) as opposed to an overall decreasing level of turnover. This pattern was also somewhat present in uninfected macrophages (Figure 2D) and could be due to inherent time-dependent autophagic regulation. In RAW 264.7, we were again surprised by negative values of turnover seen in the uninfected cells at 24, 48 and 72 h (Figure 2E), thus establishing that Bafilomycin treatment did not always lead to the accumulation of autophagy associated proteins. From this and previous experiments (Figure 1 and Figure 2), we concluded that LC3B degradation was most efficiently occurring at 4 and 48 h and there was a concomitant increase in induction over time. We were, however, not sure whether this induction was in response to LAP or autophagosomes.

### 3.3. THP-1 and RAW 264.7 Macrophages Exhibit Decreasing Levels of Autophagosomal Degradation over Time

**Figure 3:**
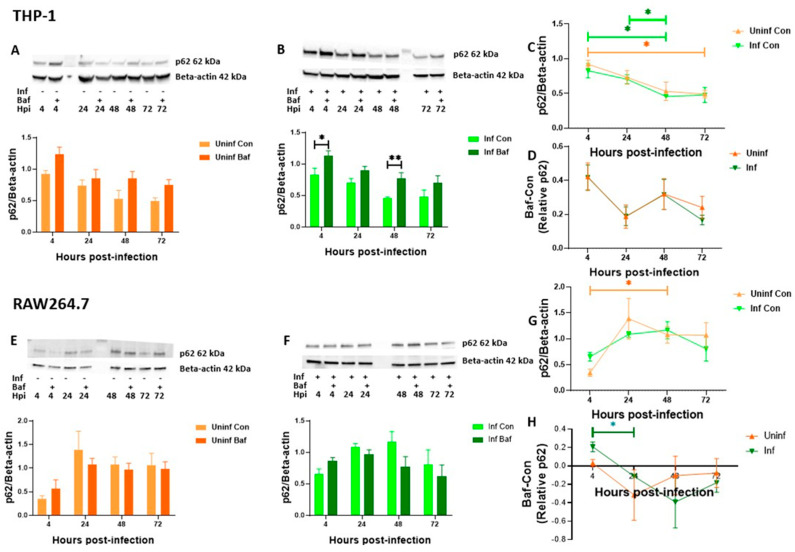
**p62 protein levels and turnover in THP-1 and RAW 264.7 macrophages over time.** THP-1 and RAW 264.7 macrophages were infected with M. tb for 4, 24, 48 and 72 h. At each time point, proteins were extracted, and western blot was performed for p62. Expression levels for uninfected THP-1 and RAW 264.7 macrophages are depicted in (**A**,**E**) respectively while infected macrophages are depicted in (**B**,**F**). Turnover was calculated by subtracting control (Con) values from Bafilomycin treated (Baf) western blot expression values for thhe respective proteins at different time points. Control only expression is depicted in (**C**,**G**) while turnover values are depicted in (**D**,**H**). Data are represented as mean ± SEM from at least three independent experiments. (* *p* ≤ 0.05, ** *p* < 0.01, Multiple *t*-test (**A**,**B**,**E**,**F**), Two-way ANOVA with Tukey test for multiple comparison (**C**,**D**,**G**,**H**). Uninf: Uninfected samples, Inf: Infected samples, Con: DMSO vehicle control, Baf: Bafilomycin A1 lysosomal inhibitor. X axis indicates time in hours and y axis indicates arbitrary units of expression of turnover values of the target protein relative to Beta-actin expression.

Next, we investigated p62 levels with the same parameters to specifically measure the changes in damaged-induced autophagy activity over time. Interestingly, both uninfected and M. tb-infected THP-1 macrophages displayed a significant decrease in p62 protein expression over time (Figure 3A–C). 

For RAW 264.7 macrophages, an increase in p62 expression after 4 h and then a gradual decrease was observed (Figure 3E–G). While the course of infection did not significantly affect p62 levels in RAW 264.7 cells, the significant decrease up until 48 h observed in THP-1 cells did not correspond to increasing levels of turnover. The cells rather maintained the alternating pattern similar to that observed for LC3B (Figure 2D and Figure 3D), with significant turnover seen at 4 and 48 h (Figure 3B). It was now clear that the significant turnover at 4 and 48 h, also seen in LC3B, was that of autophagosomal degradation. In RAW 264.7 cells, as also seen for LC3B (Figure 2E), the macrophages exhibited negative turnover values at later time points in both uninfected and M. tb-infected macrophages (Figure 3E,F). Taken together, these results highlight that significant autophagosomal degradation was occurring in M. tb infected macrophages at 4 and 48 h. This was ascertained from significant LC3B turnover observed in THP-1 and RAW 264.7 macrophages and p62 turnover observed in THP-1 macrophages. The relatively stable levels of p62 turnover observed at 48 to 72 h (Figure 3D,H) and the lack of significant turnover at 72 h (Figure 2F and Figure 3F), suggests that there was a dysfunction in the autophagy pathway in these M. tb-infected macrophages at 72 h. Additionally, we confirmed that the expression patterns of these proteins were not sufficient to indicate the autophagic activity of the cells. Turnover data and its changes over time provided more insights. From the observation that infected macrophages followed similar trends compared to their uninfected counterparts with significant changes at specific time points, suggested that macrophages possess a rapid feedback mechanism that remains conserved during M. tb infection. We also observed switching to Beta-actin as a loading control did not make positive the negative turnover values previously observed for p62 in THP-1 macrophages (Figure 1B and Figure 3D ). However, we still could not explain the reason(s) for the negative LC3B and p62 turnover values seen in RAW 264.7 cells.

### 3.4. LC3B Puncta Count, and Area Are Increased in Bacteria-Containing RAW 264.7 Macrophages

**Figure 4:**
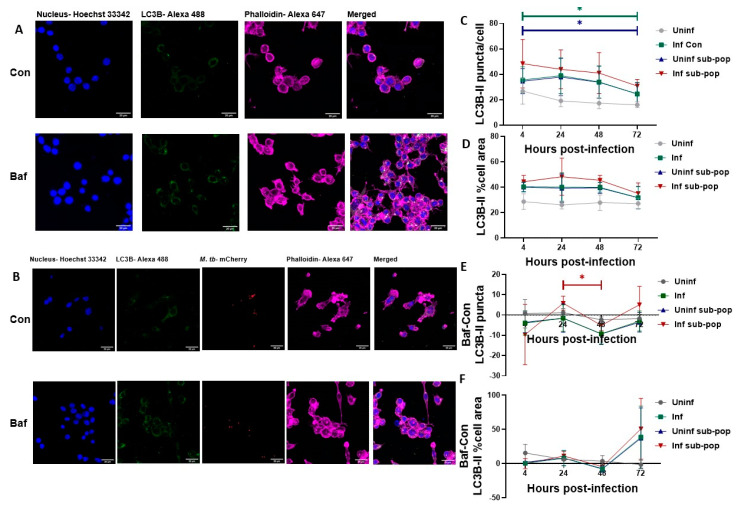
**LC3B puncta level and turnover in RAW 264.7 macrophages.** RAW 264.7 macrophages were infected with M. tb for 4, 24, 48 and 72 h. At each time point, cells were fixed, permeabilized and immunostained for LC3B. Representative confocal images of uninfected macrophages at 4 h are shown in (**A**) while those of infected macrophages at 4 h post-infection are shown in (**B**). (**C**) shows the number of LC3B puncta per cell for the indicated sample groups. (**D**) shows the percentage cell area occupied by LC3B puncta. (**E**) shows the turnover of LC3B puncta count and (**F**) shows the turnover of LC3B percentage cell area. Data are represented as mean ± SEM from three independent experiments. (* *p* ≤ 0.05 Two-way ANOVA with Tukey’s test for multiple comparison). Turnover was calculated by subtracting Control (Con) puncta count (**A**) or percentage cell volume (**B**) from their respective Bafilomycin treated (Baf) values. Scale bar is 20 µm. Uninf: Uninfected samples, Inf: Infected samples, sub-pop: subpopulations.

To further decipher the reason for negative turnover, we conducted confocal microscopy of these RAW 264.7 cells, to implement a single cell analysis pool size approach [60]. Here, as opposed to measuring the overall protein signal intensity, we counted the total number of LC3 positive puncta and measured the percentage cell area occupied by these puncta as additional metric (Figure 4A,B). 

For this set of experiments, we also aimed to resolve bacteria-specific events such as phagosomal damage from events arising from an infectious environment. We achieved this by fluorescently discerning the pool of M. tb exposed cells into infected subpopulation (bacteria-containing) and uninfected subpopulations (See Table S1 for cell counts per population group). After resolving, LC3B puncta were higher in count and percentage cell area in the infected subpopulations compared to the uninfected subpopulation at all time points, with the uninfected healthy controls having the lowest count and cell area among all population groups (Figure 4C,D). Their levels also remained relatively constant over time, similar to those assessed through western blot analysis (Figure 2G). LC3B puncta count turnover interestingly remained negative while cell area was mostly positive (Figure 4E,F). This distinction was not apparent from previous western blot data (Figure 2D,H). This suggested that upon Bafilomycin treatment in RAW 264.7 macrophages, LC3B puncta aggregated, thereby reducing in the count but occupying a larger cell area. Turnover of LC3B puncta count also showed a significant decrease from 24 to 48 h which was replicated in the cell area although not significant (Figure 4E,F). This high level of turnover at 24 h was not significantly different from a similar peak seen at 72 h and thus did not explain the possibility of phagosomal damage and subsequent autophagic targeting occurring at one of the time points. Examination of p62 puncta was necessary to elucidate this observation.

### 3.5. P62 Puncta Turnover in the Infected Subpopulation of RAW 264.7 Macrophages Is Highest at 48 h

**Figure 5:**
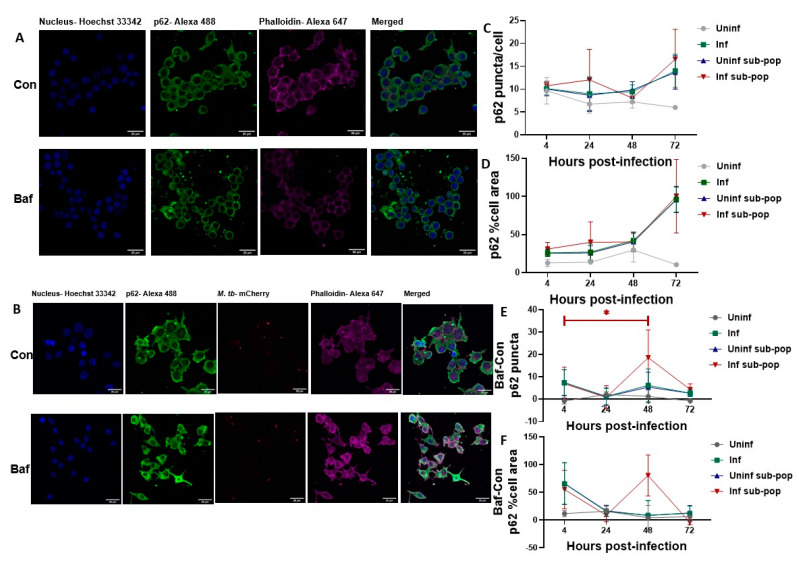
**p62 puncta level and turnover in RAW 264.7 macrophages.** RAW 264.7 macrophages were infected with M. tb for 4, 24, 48 and 72 h. At each time point, cells were fixed, permeabilized and immunostained for p62. Representative confocal images of uninfected macrophages at 4 h are shown in (**A**) while those of infected macrophages at 4 h post infection are shown in (**B**). (**C**) shows the number of p62 puncta per cell for the indicated sample groups. (**D**) shows the percentage cell area occupied by p62 puncta. (**E**) shows the turnover of p62 puncta count and (**F**) shows the turnover of p62 percentage cell area. Data are represented as mean ± SEM from three independent experiments. (* *p* ≤ 0.05 Two-way ANOVA with Tukey’s test for multiple comparison). Turnover was calculated by subtracting Control (Con) puncta count (**A**) or percentage cell volume (**B**) from their respective Bafilomycin treated (Baf) values. Scale bar is 20 µm. Uninf: Uninfected samples, Inf: Infected samples, sub-pop: subpopulations.

P62 puncta remained constant over time but increased in infectious conditions at 72 h though this was not significant (Figure 5A–D).

Compared to LC3B puncta turnover (Figure 4E,F), p62 puncta which more specifically are degraded within autophagosomes showed the highest turnover value at 48 h in the infected subpopulation. This turnover of the p62 puncta count was significantly higher from a similar peak seen at 4 h (Figure 5E). Turnover of the puncta area also showed a peak at 48 h that was exclusive to the infected subpopulation (Figure 5F). These clear observations, combined with previous p62 western blot data suggested that though significant autophagic degradation occurs at 4 and 48 h, the highest bacteria-specific, damage-induced autophagic degradation occurred at 48 h post-infection. 

### 3.6. Fold Change of M. tb Bacilli Is Highest at 48 h Post-Infection

**Figure 6:**
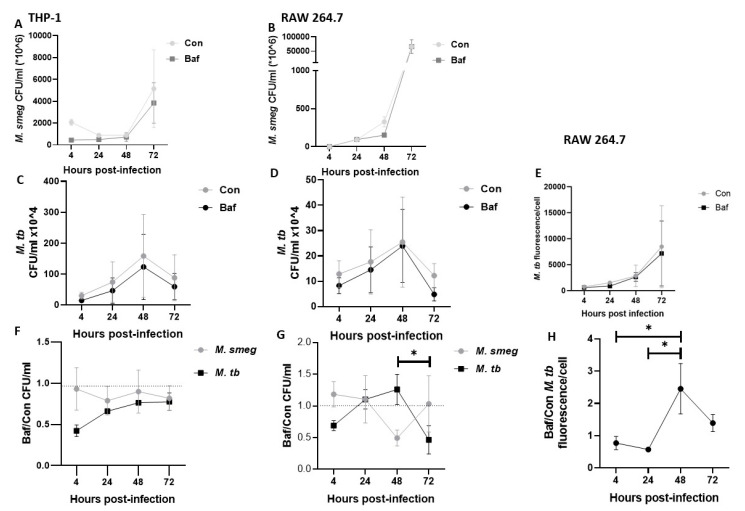
**Fold change of *M. smegmatis* and M. tb in THP-1 and RAW 264.7 macrophages.** (**A**,**B**) show *M. smegmatis* growth over time in THP-1 and RAW 264.7 macrophages respectively, while (**C**) shows M. tb growth in THP-1 macrophages and (**D**,**E**) show M. tb growth in RAW 264.7 macrophages over time. (**F**) shows bacteria fold change in THP-1 macrophages while (**G**,**H**) show bacterial fold change in RAW 264.7 macrophages. Fold change was calculated by dividing Bafilomycin mycobacteria count by their respective control samples before averaging. CFU/mL data are not comparable between THP-1 and RAW264.7 as different macrophage counts were used when seeding cells as RAW264.7 are smaller in size. Data are represented as mean ± SEM from four independent experiments. (* *p* ≤ 0.05 Two-way ANOVA with Tukey’s test for multiple comparison).

Lastly, to confirm our findings of bacteria-specific autophagic activity occurring mostly at 48 h post-infection, we examined the fold change of M. tb bacilli. This we achieved by calculating the fold change of M. tb colony forming units and fluorescence intensity. We first established the growth patterns of the non-pathogenic *Mycobacterium smegmatis* (Figure 6A,B) and M. tb (Figure 6C–E) to be similar in both THP-1 and RAW 264.7 macrophages. 

Mycobacterium tuberculosis fold change showed a steady increase from 4 h up until 48 h (Figure 6F–H) and then decreased significantly in RAW 264.7 macrophages (Figure 6G), strikingly different from the pattern observed in the non-pathogenic *Mycobacterium smegmatis*. The highest level of M. tb bacterial fold change observed at 48 h was also significantly higher than at 4 and 24 h as measured by bacterial fluorescence intensity (Figure 6H). Taken together, these results suggested phagosomal damage and autophagy activation occurred mostly at 48 h post M. tb infection 

## 4. Discussion

It is well documented that exogenously induced autophagy plays an important role in the clearance of M. tb in host cells [23]. However, the dynamics of autophagy activity under basal conditions and upon infection as well as and its impact on mycobacterial viability over time is largely under-studied. Most M. tb studies have either explored this process using a limited number of autophagy markers [3,36,37,38,39,40,41,42,43,44] or studying one time point only [3,38,39,40,41,42,45,46]. The present study focused on documenting autophagy induction and turnover in different infection conditions over time, also using different analytical tools. This present study aimed to provide a comprehensive analysis of autophagy activity under basal conditions in macrophage cell lines and highlight potential time point(s) of high autophagic activity. 

Upon detection of bacterial infection, infected macrophages recognize this intrusion and initiate an autophagic stress response [61]. This stress response is characterized by a combination of macroautophagy whereby random cytoplasmic contents are degraded to provide the energy boost needed to fight off the infection and selective autophagy where specific bacteria and their secreted virulent proteins are targeted for degradation. While M. tb could induce macroautophagy to provide host-derived nutrients for its growth, selective autophagy, on the other hand, is detrimental to M. tb’s survival. The selective recognition and tagging of the invading bacteria by ubiquitination links bacterial pathogens to the autophagy machinery through receptor proteins such as p62. In experiments where we compared the turnover of uninfected and infected THP-1 macrophages, we found that p62 and RAB7 but not LC3B had increased turnover values in infected macrophages at all time points (Figure 1). If one considers that all p62 labelled cargo available in the cytoplasm will eventually become LC3B positive and thereafter recruit RAB7, two scenarios can be proposed. One, levels of p62 and RAB7 turnover are very low in uninfected macrophages, thereby amplifying the levels observed in infected macrophages. Secondly, newly formed p62 and RAB7 compartments merge with basal LC3B compartments, resulting in similar levels of LC3B turnover in infected macrophages. We speculate that both scenarios account for the observations in this study [31].

To elucidate autophagy progression in this study, we assessed autophagy turnover in THP-1 and RAW 264.7 macrophages. As expected, we observed notable differences in these two cell lines. For one, RAW 264.7 macrophages often presented with negative turnover values. While the reason for this is not clear in literature, we hypothesize these negative turnover values arise because of low autophagy turnover and increase in puncta size upon Bafilomycin treatment. Data from a report using both THP-1 and RAW 264.7 in assessing autophagy flux showed THP-1 macrophages indeed have a higher autophagy flux compared to RAW 264.7 macrophages [49]. Microscopic analysis of LC3B and p62 puncta in RAW 264.7 cells also showed that although LC3B puncta were reduced in count upon Bafilomycin treatment in most instances, an increase in relative cell volume was seen in these puncta suggesting fusion or aggregation of puncta upon Bafilomycin treatment. Also, while cell growth is halted in THP-1 macrophages after PMA differentiation and seeding, RAW 264.7 cells continue to proliferate. We, therefore, reduced the FBS concentration in the RAW 264.7 cellular medium to reduce the rate of proliferation of these cells through the time points being studied. With 10% FBS, RAW 264.7 cells have been shown to have a doubling time of 11–12 h [62]. In our study using 2.5% FBS, we only observed visible division of cells at about the 48-h time point. Though we reduced the FBS concentration in these cells, we also daily replenished the cell medium in both uninfected and infected macrophages to avoid the cells going into starvation, viability was also not affected (Figure S1). This slower rate of proliferation also likely reduced basal autophagy flux in these cells [63]. It is also important to mention again that the decreased expression of proteins we observed upon Bafilomycin treatment were not significant (Figure 2E and Figure 3E,F). Despite these explanations, our experiments did not directly test for differences between the two cells, but we rather discussed the combined patterns observed in both cells.

Within the pool of infected macrophages, the common observations across all experiments were the significant increases in protein expression upon Bafilomycin treatment at 4 and 48 h. This suggests significant autophagy activity occurring at the 4- and 48-h time points. 

At 4 h post-infection, it is expected that macrophages would mount a strong autophagy response to the infection. Parkin, the E3 ligase and ubiquitin have both been observed to colocalize with M. tb at this early time point of infection suggesting some level of autophagy activation [18]. At this point, we envisage LAP, as well as the selective degradation of virulent proteins and possibly M. tb bacilli, are induced. Interestingly, murine macrophages induce LAP to a greater degree than human macrophages. It was observed that between 40% and 80% of the total phagosomes in mouse cells but only 5% to 10% in human cells were LC3-associated and lived longer than 4 h [64,65]. One of the earliest studies to investigate LAP in M. tb infection showed that attenuating the expression of the mycobacterial protein CpsA increased colocalization of M. tb with the lysosome [47]. This provided evidence for the involvement of the mycobacterial CpsA protein in LAP evasion. However, although murine macrophages induce LAP to a greater extent than human macrophages, M. tb is still capable of inhibiting LAP-lysosome targeting and therefore one would expect less LC3B turnover in RAW 264.7 macrophages. This would also explain why more significant LC3B turnover was seen in THP1 cells compared to RAW 264.7 cells (Figure 2D,H).

As infection progressed to 48 h, the combined inherent heightened autophagy activity of the macrophages (as observed in uninfected cells) and the presence of the intracellular pathogen-induced autophagy activity even further (Figure 2). It is also possible that a significant event in the infection lifecycle such as cytosolic translocation triggered the significant Bafilomycin response at 48 h. These significant differences were, however, not seen in the microscopy data possibly because puncta count, and puncta relative cell volume were measured separately. However, it was evident that puncta count, and relative cell volume were higher in infected macrophages compared to their uninfected counterparts (Figure 4 and Figure 5). 

The response of the subpopulations was solely provided by immunofluorescence imaging data from RAW 264.7 macrophages. This allowed us to differentiate bacilli-specific events from general infection events. Within the uninfected subpopulations, we observed very similar responses to those of the general pool of infected macrophages due to the higher percentage of uninfected subpopulations within the general pool.

Lastly, within the infected subpopulation, we observed a significant increase in p62 puncta turnover from 4 to 48 h (as measured by puncta count). This provided compelling evidence that a stronger event, triggered by M. tb bacilli was inducing significant autophagy activity at 48 h compared to 4 h. The similar level of autophagy turnover observed in the uninfected subpopulation and infected subpopulation of infected macrophages at the other time points of 4, 24 and 72 h was an interesting observation, which makes one curious about the nature of the degraded cargo in the uninfected subpopulation. Firstly, we hypothesize that apart from bacteria-specific degradation at 48 h, autophagy degradation is reduced at other time points in the infected subpopulations. Also, both subpopulations degrade (to a higher degree than uninfected macrophages) long-lived proteins and damaged organelles. This heightened degradation is necessary to provide the extra energy needed after macrophages are signaled by cytokines/chemokines from neighboring cells (for uninfected subpopulations), intracellular bacterial effector proteins (for infected subpopulations) and extracellular bacteria effector proteins (for both macrophage groups) of the ongoing bacterial evasion.

The present study is the first to use fluorescence-based discernment to distinguish the pool of infected macrophages, i.e., uninfected and infected subpopulations and thereafter perform autophagy analyses. This method of analyzing the subpopulations separately was advantageous in comparison to present methods of analyzing the pool of infected macrophages. This approach enables researchers to study changes in neighboring, no bacilli-containing cells and how they respond to the infection environment despite having no internalized bacterium. Such results will aid studies investigating macrophage priming for impending infection. This also allows for investigating intracellular bacteria-specific events. In this study, this approach enabled us to identify bacteria-specific events at 48 h which were previously masked in the pool of infected macrophages. 

To corroborate the p62 turnover results, we investigated the fold change of M. tb bacilli. Of note, we obtained correlating results with both the measurement of bacteria fluorescence and counts of colony-forming units. Both techniques in RAW 264.7 macrophages revealed the highest fold change at 48 h post-infection. For THP-1 macrophages, CFU enumeration similarly showed a steady increase in fold change from 4 to 48 h before plateauing at 72 h. This confirmed that the highest degradation of intracellular M. tb by selective autophagy occurs at 48 h post-infection (Figure 6F–H).

Among other inducers of autophagy, damaged or dysfunctional phagosomes are the most probable reason why significant autophagy turnover could occur at 48 h in M. tb-infected macrophages [16,17,29,30]. Interestingly, two studies have reported the highest cytosolic occupation of M. tb at 48 h post-infection. One was observed in granulocyte-macrophage colony-stimulating factor (GM-CSF) differentiated macrophages when comparing 2, 24 and 48 h [66] and the other in human induced pluripotent stem cell-derived macrophages when comparing 2 h versus 48 h [67].

Access to nutrients in the cytosol and to neighboring uninfected cells has been proposed to be one of the main reasons for M. tb’s cytosolic translocation [17,29,66]. Consistent with this, Bafilomycin pre-treatment prior to infection, which inhibits lysosomal degradation and thus the availability of nutrients, was shown to enhance phagosomal rupture in M. tb-infected macrophages [15]. Also, the mycobacterial ESX-1 secretion system [16] and the Phthicerol dimycocerosate previously linked with virulence has been implicated in inducing phagosomal rupture [17,30,66,68,69]. The ruptured phagosomes are then transiently associated with ubiquitin molecules and degraded via the autophagy machinery [29]. Cytosolic M. tb, on the other hand, can evade autophagy targeting by removing p62 labelling and proceeding to form cords to aid survival [24]. Successful bacterial clearance after cytosolic translocation is then dependent on how successful the autophagy machinery degrading the ruptured phagosome can capture translocating M. tb. It is proposed that bacilli near the ruptured phagosomes are more likely to be captured and degraded, however this process may be cyclical if M. tb damages the autophagosome membrane [24]. After cytosolic translocation, a cascading series of necrotic macrophage cell death events have been observed, likely as a result of the burden of intracellular cords being formed and a pathogenic mechanism by M. tb to ensure cell to cell spread [16,66,70].

The present study reported turnover as the change in autophagy protein abundance with and without lysosomal inhibition, extrapolating this as a measure of autophagy flux. A previous study has more accurately characterized and described autophagy flux to depict the rate of turnover of autophagy cargo [60]. In those experiments, live, real-time quantification of cargo and transition time at a steady state were assessed in mouse embryonic fibroblasts [60]. This differs from what this study assessed and we acknowledge this as a limitation. The lack of a live cell imaging microscope in our Biosafety Level III laboratory limited our ability to measure and quantify real-time autophagy activity. We acknowledged that the results obtained in this study should be interpreted with this in view. Also, a direct comparison of the mycobacteria killing ability of THP-1 versus RAW264.7 was not tested. Proliferation of RAW264.7 macrophages and their smaller size made direct comparison challenging. This study rather focused on the combined patterns of autophagy induction and mycobacteria killing seen in both cell lines.

**Figure 7:**
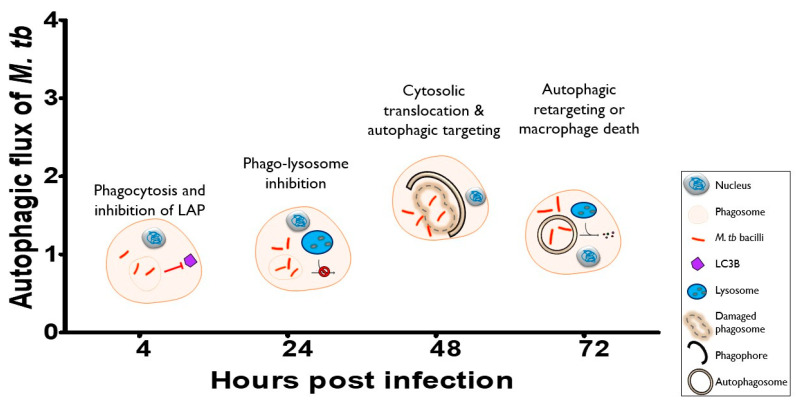
Proposed summary of autophagy pathway progression in M. tb-infected macrophages. At the start of the infection, macrophages phagocytose M. tb and induce LAP while M. tb inhibits LAP to avoid killing. At 24 h, M. tb is majorly inhibiting phago-lysosome fusion while replicating in the phagosome. At 48 h, the highest level of autophagic turnover is observed, correlating with cytosolic translocation of M. tb. At 72 h, macrophages either are able to mount another autophagic response to target cytosolic bacilli that successfully translocated or are overburdened and die off. LAP: LC3-associated phagocytosis.

Autophagy progression remains an understudied area of research in disease pathogenesis. In M. tb infection, temporal differences are still debated and are not clearly elucidated. This study provided a strong background on autophagy progression in the THP1 and RAW264.7 macrophage cell lines. Correlating our results with literature findings, we propose a summary for the infection time course in Figure 7. 

At the start of infection, phagocytosis and the concurrent inhibition of LC3-associated phagocytosis are the major events occurring at this 4-h time-point (Figure 1; [47]), As the infection progresses, LC3B turnover levels decreased at 24 h post-infection and thereafter increased at 48 h post-infection (Figure 2). Correlating these with increased bacterial growth from 24 to 48 h post-infection (Figure 6), phago-lysosome inhibition most likely occurs at the 24-h time point. After this, a heightened level of p62 turnover was observed, especially in the infected macrophages subpopulation indicating high levels of damaged-induced autophagic targeting (Figure 5 and Figure 6). We postulate cytosolic translocation induces autophagic targeting at this 48-h point [17,24,68]. After cytosolic translocation of M. tb, infected cells are observed to either induce autophagic retargeting [24] or undergo a cascading series of necrotic cell death [54,66,71], we postulate these mostly happen at 72 h post-infection. Our observations of increasing p62 puncta but reduced p62 and bacilli fold change at 72 h (Figure 5 and Figure 6) support unsuccessful autophagic retargeting.

In this study, the relative expression levels and turnover of the autophagy proteins- LC3B and p62 differed in uninfected and M. tb-infected macrophages at different time points of the M. tb infection lifecycle. We suggest laboratories with live-cell imaging capabilities in their BSL3 facilities to include a measure of the rate of protein degradation and to compare it over a time course. Moreover, LC3B and p62 measurements could be explored to identify patients most likely to benefit from autophagy-related anti-tuberculosis therapy. Promising results already exist in this regard where a defect in autophagy induction correlated with poor TB disease outcomes [72]. Supporting this notion, autophagy proteins (LC3B and p62) are currently being investigated as prognostic markers in cancer [73,74,75,76,77,78,79,80]. After phagosomal damage, it is still unknown which M. tb proteins and phagosomal membrane proteins become ubiquitinated leading to autophagy activation. These could either be investigated by assessing global protein turnover with mass spectrometry or putative targets could be assessed for co-immunoprecipitation with E3 ligases of interest. Translation of this work could also involve an investigation into the biomarker potential of the markers utilized in the study. One approach may be to investigate the responses of these markers in whole blood [81,82] or monocyte-derived macrophages from TB patients. Future studies could also investigate the membrane proteins of these different compartments as these may present as targets of the autophagy machinery. 

## 5. Conclusions

Our aim for this research was to track autophagy activity at different time points in M. tb-infected macrophages and their uninfected controls. We showed highest autophagy turnover occurs at 48 h in M. tb-infected macrophages. This correlates with reports of the highest cytosolic occupation of M. tb in host cells at 48 h [66] and earlier observation of cytosolic translocation at 48 and 72 h [17]. Our results also support the current use of autophagy markers to investigate the autophagy machinery and response. However, we encourage researchers to expand the portfolio of markers and time points studied. LC3B is the most common autophagy marker used, however it does not differentiate between LAP where LC3B is recruited to intact phagosomes and selective autophagy where selective substrates such as damaged phagosomes or cytosolic bacteria are targeted. P62, on the other hand, selectively binds to ubiquitin on damaged substrates. It is therefore advisable to rather use p62 when studying selective autophagy in the context of bacterial infections. Our results have also shown the dynamic changes in the autophagy response over time. We suggest measurement of autophagic response should not be based on one time point alone. Lastly, we explored the use of puncta count as well as the measurement of puncta relative cell volume. Both parameters resolved the microscopic measurement of autophagy events and provided additional information that was biologically relevant. For example, we uncovered infection conditions and or time points that induced puncta fusion/aggregation as opposed to an increase in puncta count. This difference is missed in western blot analysis and underscores microscopic analysis as an important technique in autophagy studies.

This research characterized autophagy progression and identified 48 h as the time point with the highest bacteria-specific autophagic turnover in M. tb-infected macrophages. This provides a strong background for future research in M. tb host-pathogen interactions and host-directed anti-TB therapy. Characterization of the time points also affords more targeted research endeavors and the development of more effective autophagy-tuberculosis therapies.

## Data Availability

The microscopy data reported in this study can be accessed here: 10.5281/zenodo.7273259.

## Supplementary Materials

The following supporting information can be downloaded at: https://www.mdpi.com/article/10.3390/pathogens12010024/s1, Table S1: Cell counts per infection population group; Figure S1: Bafilomycin treatment of 100 nM for three hours does not decrease cell viability in THP-1 and RAW264.7 macro-phages.
