# Supplementary material for: Measurement of Autophagy Activity Reveals Time-Dependent, Bacteria-Specific Turnover during Mycobacterium tuberculosis Infection
Source: Pathogens. 2022 Dec 23;12(1):24. doi: 10.3390/pathogens12010024 (PMC9864524; doi:10.3390/pathogens12010024)
Supplement: Supplementary file 1 [file pathogens-12-00024-s001.zip › pathogens-2071610-supplementary.pdf]

Supplementary Table S1 (Table S1)

Table S1. Cell counts per infection population group.

No. of cells counted (LC3B-II)	4hrs		24hrs		48hrs		72hrs		Total
	Con	Baf	Con	Baf	Con	Baf	Con	Baf	
Uninfected	262	284	421	640	600	918	812	779	4716
Infected	299	274	235	291	338	420	287	513	2657
Uninfected sub-pop	277	255	192	238	310	377	275	453	2377
Infected sub-pop	22	19	43	53	28	43	12	60	280
Total	860	832	891	1222	1276	1758	1386	1805	10030
% bacteria-containing macrophages	7.35786	6.934307	18.29787	18.21306	8.284024	10.2381	4.181185	11.69591	10.5382
**No. of cells counted (p62)**	**4hrs**		**24hrs**		**48hrs**		**72hrs**		**Total**
	**Con**	**Baf**	**Con**	**Baf**	**Con**	**Baf**	**Con**	**Baf**	
Uninfected	269	360	423	514	648	790	652	946	4602
Infected	231	314	259	345	375	443	495	480	2942
Uninfected sub-pop	214	293	248	318	331	385	464	456	2709
Infected sub-pop	17	21	11	27	44	58	31	24	233
Total	731	988	941	1204	1398	1676	1642	1906	10486
% bacteria-containing macrophages	7.359307	6.687898	4.247104	7.826087	11.73333	13.09255	6.262626	5	7.919782

Supplementary Figure S1 (Figure S1)

A

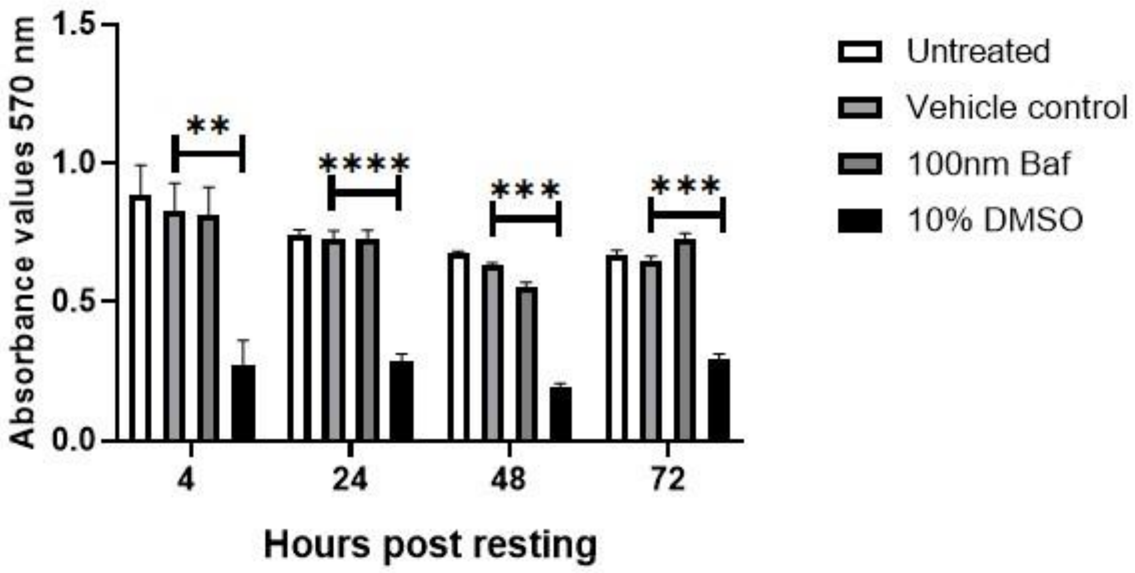

B

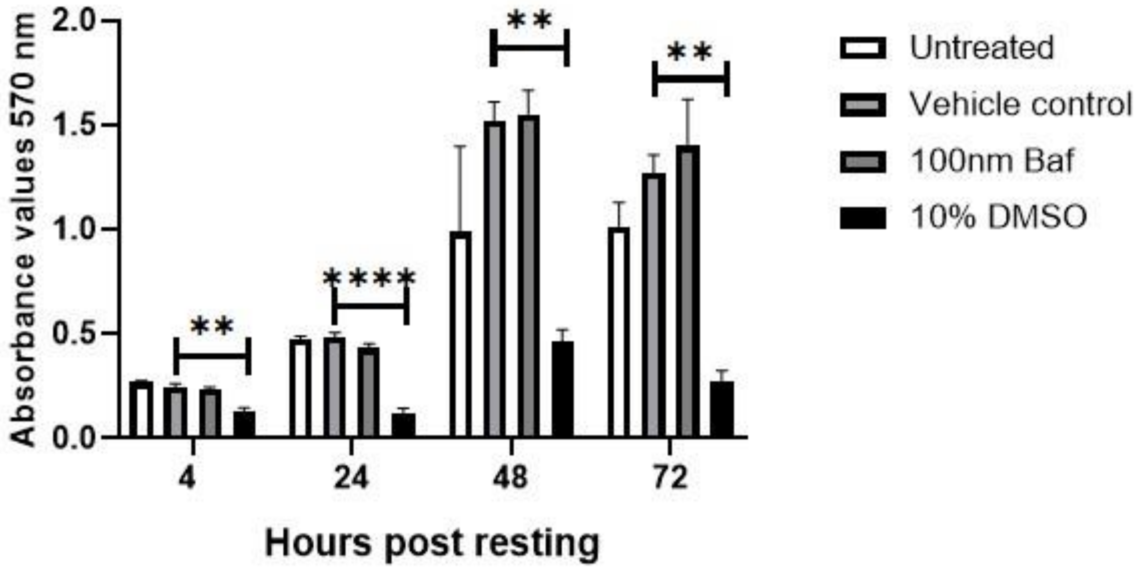

Figure S1. Bafilomycin treatment of 100 nM for three hours does not decrease cell viability in THP-1 and RAW264.7 macrophages. THP-1 (**A**) and RAW 264.7 (**B**) were treated with 100 nM Bafilomycin for three hours prior to each time point. Macrophages were then subjected to MTT cell viability test at 0.25 mg/mL MTT. Data shows Bafilomycin treatment had no negative effect on cell viability on these macrophages. ** $p < 0.01$, *** $p < 0.001$, **** $p < 0.0001$.
